# Angiotensin Converting Enzyme Inhibition Reduces Cardiovascular Responses to Acute Stress in Myocardially Infarcted and Chronically Stressed Rats

**DOI:** 10.1155/2014/385082

**Published:** 2014-06-19

**Authors:** A. Cudnoch-Jedrzejewska, K. Czarzasta, L. Puchalska, J. Dobruch, O. Borowik, J. Pachucki, E. Szczepanska-Sadowska

**Affiliations:** ^1^Department of Experimental and Clinical Physiology, Medical University of Warsaw, Ulica Pawinskiego 3c, 02-106 Warsaw, Poland; ^2^Department of Internal Medicine and Endocrinology, Medical University of Warsaw, Ulica Banacha 1a, 02-097 Warsaw, Poland

## Abstract

Previous studies showed that chronically stressed and myocardially infarcted rats respond with exaggerated cardiovascular responses to acute stress. The present experiments were designed to elucidate whether this effect can be abolished by treatment with the angiotensin converting enzyme (ACE) inhibitor captopril. Sprague Dawley rats were subjected either to sham surgery (Groups 1 and 2) or to myocardial infarction (Groups 3 and 4). The rats of Groups 2 and 4 were also exposed to mild chronic stressing. Four weeks after the operation, mean arterial blood pressure (MABP) and heart rate (HR) were measured under resting conditions and after application of acute stress. The cardiovascular responses to the acute stress were determined again 24 h after administration of captopril orally. Captopril significantly reduced resting MABP in each group. Before administration of captopril, the maximum increases in MABP evoked by the acute stressor in all (infarcted and sham-operated) chronically stressed rats and also in the infarcted nonchronically stressed rats were significantly greater than in the sham-operated rats not exposed to chronic stressing. These differences were abolished by captopril. The results suggest that ACE may improve tolerance of acute stress in heart failure and during chronic stressing.

## 1. Introduction

Prolonged activation of the renin-angiotensin system (RAS) plays a pivotal role in the pathogenesis of cardiovascular diseases [1, 2]. Introduction of angiotensin converting enzyme 1 inhibitors (ACEI) and angiotensin AT1 receptor blockers (AT1RB) into the treatment of cardiovascular diseases markedly reduced morbidity and mortality of cardiovascular patients [3–5]. Although large clinical trials did not indicate significant differences between the effectiveness of ACEI and effectiveness of AT1RB [6], there were also reports showing that some patients manifest preference to ACEI while others respond better to AT1RB or require supplementary treatment [7, 8]. Thus far, the reasons of individual differences in preference of AT1RB or ACEI are not fully recognized. One of the causes of individual differences in effectiveness of AT1 receptors blockers and ACE inhibitors in the cardiovascular diseases might result from different role of these compounds in the regulation of cardiovascular reactions to stress.

In recent years, evidence has been provided that angiotensin II (Ang II) may be an important stress hormone. Therefore, Yang et al. [9] reported that rats exposed to a short-lasting compulsive water swim or to prolonged stressing (5-day exposure to low ambient temperature) manifest significantly higher concentration of Ang II in the brain, adrenals, heart, vessels, and blood than the control animals. In addition, several groups of investigators provided evidence that angiotensin II and angiotensin AT1 receptors play an essential role in the stimulation of neuroendocrine responses to stress [10–13]. With regard to the regulation of blood pressure, Saiki et al. [14] reported that the blockade of AT1 receptors in the brain by intracerebroventricular (ICV) administration of losartan or saralasin reduces the cardiovascular responses to immobilization stress, whereas De Matteo et al. [15] and Mayorov and Head [16] have found that the blockade of AT1 receptors in the rostral ventrolateral medulla (RVLM) and dorsomedial hypothalamus with candesartan or losartan reduces significantly the pressor response to 7-minute exposure to air jet stressor in rabbits. Furthermore, the studies of Zhang et al. [17] and Cudnoch-Jedrzejewska et al. [18, 19] revealed that the blockade of AT1 receptors with losartan abolishes elevation of cardiovascular responses to stress in rats with myocardial infarction.

In the present study we tested the hypothesis that cardiovascular responses to stress might also be significantly influenced by orally applied ACE inhibitors. The rationale to address this question was based on the following premises. Firstly, the spectrum of ACE inhibitors action markedly differs from that of AT1RB [7, 20]. Angiotensin converting enzyme inhibitors abolish the formation of Ang II, preventing thereby activation of both AT1 and AT2 receptors, while AT1RB selectively interferes with the stimulation of AT1 receptors. In addition, ACE interferes with the destruction of kinins [21]. Secondly, chronic stressing is frequently an unavoidable attribute of everyday life and it was reported that it might aggravate the cardiovascular pathology [22]. Therefore, in this investigation, we aimed to elucidate whether oral administration of ACE inhibitors may effectively reduce cardiovascular responses to acute stress in the postinfarct heart failure, during chronic stress and during combination of these two challenges. In order to solve this question we compared effects of administration of captopril, which effectively inhibits ACE1 in rats [23, 24], on the cardiovascular responses to acute stress in infarcted and sham-operated rats, and in the rats exposed either to chronic stressing alone or to chronic stressing combined with myocardial infarction.

## 2. Material and Methods

### 2.1. Animal Husbandry

Male Sprague Dawley rats (SPRD/Möl/Lod) were used as experimental animals. They were obtained from the Department of Animal Breeding and kept under 12 h light/12 h dark rhythm (light on at 6:00 a.m.) and in a room with regulated temperature (range 22–25°C). The rats were fed standard rat chow containing 0.3% sodium chloride (NaCl) and had free access to water. All experimental and surgical procedures described below were approved by the Local Ethical Committee on Animal Research and conducted in accordance with the international/EU guidelines and regulations on the use and care of laboratory animals.

### 2.2. Surgical Procedures

All surgical procedures were carried out under barbiturate anesthesia (pentobarbital, Biowet, Puławy, 5 mg/100 g of body wt, i.p.). Immediately after surgery, the rats were given an analgesic (buprenorphine 3 *μ*g/100 g of body wt, i.p.; 2 times daily for 2-3 days) and antibiotic (penicillin, Polfa 10,000 IU/100 g of body wt, i.m.) and were placed in their own home cages.

At an age of 8–10 weeks, the rats were subjected either to a permanent left coronary artery ligation or to sham surgery. The coronary ligation was performed according to our own modification [25] of the surgical procedure described by Selye et al. [26]. Briefly, the heart was exteriorized through a surgical incision made between the 4th and 5th intercostal space while ventilation of the lungs was maintained by frequent administration of air jet puffs by means of a small rubber balloon connected to the rat's nose by means of a plastic tube. The left coronary artery was permanently ligated with a suture thread (Ethicon 6.0). The heart was placed back into the thoracic cavity and the wound was closed with surgical sutures (Ethicon 4.0). The rate of survival from the surgery was 48%. In the sham-operated rats the coronary artery was not ligated—instead the pericardium was touched with a needle. The rate of survival of the sham-operated rats was 95%.

Five weeks after the thoracic surgery, rats at the age of 13–15 weeks had an arterial line implanted for the measurement of mean arterial blood pressure (MABP) and heart rate (HR). The line consisted of an intra-arterial portion, made from a 3.5–4.0 cm tubing (i.d., 0.12 mm; o.d., 0.25 mm), and the external portion (i.d., 0.25 mm; o.d., 0.4 mm), made from a polyvinyl tubing (Scientific Commodities Inc.). The arterial catheter was inserted into the aorta through the femoral artery so that its end was located 2 cm below the renal arteries. The external tubing was tunnelled under the skin and exteriorized on the neck. The catheter was filled with 0.9% physiological NaCl, containing 500 U/mL of heparin, and closed with a stopper.

### 2.3. Course of Experiments and Experimental Groups

#### 2.3.1. Course of Experiments

**Figure 1:**
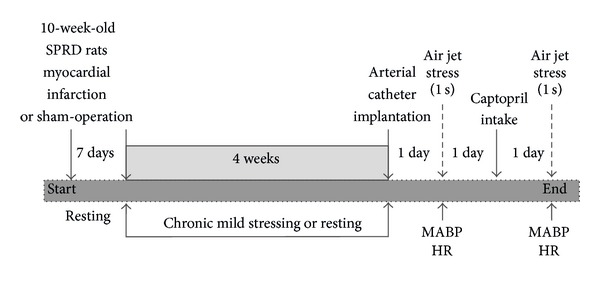
Experimental procedures. MABP: mean arterial blood pressure; HR: heart rate.

Five weeks after the myocardial infarction or sham surgery and 48 h after implantation of the arterial catheter, the rats were divided into four experimental groups (29 rats, body weight: 327–343 g) and four supplementary groups (19 rats, body weight: 335–345 g). During the experiments, the rats remained in their home cages but food and fluid were removed. For the experimental group, each experiment consisted of two parts performed on two consecutive days (Figure 1). During part 1, the rat was connected to the arterial line and the resting MABP and HR as well as MABP and HR responses to the air jet stressor were recorded. After 30 minutes allowed for adaptation, MABP and HR were recorded continuously for 40 minutes under resting conditions and for 10 minutes after the application of the alarming stressor (Figure 1). Subsequently, the rat was disconnected from the line and offered free access to food and water containing captopril (15 mg/35 mL/24 h/rat; approximately 45 mg/kg/24 h; that is, 0.207 mmol/kg/24 h). This dose of captopril was previously found to normalize arterial blood pressure in spontaneously hypertensive rats by [24, 27]. The amount of water in the bottle corresponded to an average 24 h water intake consumed by the rats receiving the same diet [28]. In all experiments, the rats ingested the total amount of captopril contained in the drinking fluid. Measurements of the cardiovascular parameters were repeated 24 hours later during the second part of the experiment as shown in Figure 1. At the end of each experiment, the rat was anesthetized with pentobarbital and a thin catheter (i.d., 0.5 mm; o.d., 0.8 mm; Dural Plastics and Engineering, Auburn, Australia) was inserted into the left ventricle of the heart via the right carotid artery and the aortic arch. The catheter served to determine the left ventricle end-diastolic pressure (LVEDP).

#### 2.3.2. Measurements and Procedures

Blood pressure, heart rate, and end-diastolic left ventricle pressure (LVEDP) were determined by means of the blood pressure recording system (BIOPAC, MP 100, Santa Barbara, CA, USA). MABP was determined as the area under the arterial pressure curve divided by the cardiac cycle duration. Heart rate (beats/min) was calculated from the number of the systolic pressure peaks.

The cardiovascular response to acute stress was estimated using our own modification [25] of the air jet stress procedure described by Zhang et al. [17]. The air jet was blown on the top of the rat's head for 1 second via a laboratory-made device. The device consisted of a tank containing compressed air (10 atmospheres) and connected by a plastic tube (i.d., 3.0 mm) to a funnel (i.d., 41.5 mm) held 1.5–2.0 cm above the rat's head. The cardiovascular measurements were collected continuously for 10 minutes following the air jet application. The magnitude of the cardiovascular responses to the air jet stressor was evaluated by measuring the maximum increases in MABP (ΔMABPmax) and heart rate (ΔHRmax) after application of the stressor. The maximum increases in these parameters were determined by subtracting the resting values (found immediately before the application of the air jet) from the maximum values of MABP and HR recorded during the first 5 seconds after application of the air jet. The latency to the maximum increases in MABP and HR and the duration (time between the onset and the end) of the pressor and tachycardic responses were also determined.

LVEDP was determined at the end of the 2nd part of the experiment.

#### 2.3.3. Groups of Experiments

Group 1 (control) was performed on 7 sham-operated rats with the purpose of determining the effect of ACE inhibition on MABP and HR under resting conditions and during the alarming (air jet) stress in the rats that were neither infarcted nor chronically stressed.

Group 2 was performed on 7 sham-operated, chronically stressed rats in order to determine the effects of captopril on resting cardiovascular parameters and cardiovascular responses to the alarming stressor in the sham-operated rats, exposed to chronic mild stressing.

The program of chronic stressing was similar to that used by Grippo et al. [29], except for some modifications described previously [30]. Briefly, the stressing was started one week after the thoracic surgery and consisted of five sessions per week (one session/day), which was followed by two days of rest. The following procedures were applied: (1) exposure of the rat to stroboscopic lamp flashes (300 flashes/min for 5 h); (2) placing the rat's cage in an oblique position (angle 40°) for 6 h; (3) visit of another rat in the home cage of the experimental rat for 3 h (during the visit the rats were separated by a transparent barrier); (4) water deprivation for 18 h followed by access to an empty bottle for 6 h; (5) placing the rat for 4 h in a new smaller cage (30 × 30 × 30 cm), in which it was exposed to an alien smell (deodorant placed in a perforated box). During each week, the sequence of the stressing procedures was altered. After four weeks of chronic stressing, the experiment was performed according to the protocol described in Group 1. Each stressing procedure started at 10:00 a.m. The same model of mild chronic stressing was found to induce anhedonia and significant changes in blood renin and corticosterone levels ([29] and our own unpublished data).

Group 3 was performed on 8 infarcted rats and was aimed at determining the effect of captopril on resting MABP and HR and on the cardiovascular responses to air jet stress in the infarcted rats, not exposed to chronic stressing. The experimental design was the same as in Group 1.

Group 4 was performed on 7 infarcted, chronically stressed rats in order to determine the effect of captopril on resting MABP and HR and on the cardiovascular responses to acute stress during combined exposure to the postinfarct heart failure and chronic stressing. The experimental protocol and the program of stressing were the same as in Group 2.

#### 2.3.4. Supplementary Experiments

Supplementary experiments were performed to find out whether the rats could adapt to the air jet stressor when it was applied with 24 h intervals. Eight 10-week-old rats were subjected to the sham surgery (Group 5; *n* = 4 and Group 6; *n* = 4) or to a permanent coronary ligation (Group 7; *n* = 5 and Group 8; *n* = 4). Subsequently, the rats of Groups 5 and 7 were staying at rest for four weeks whereas the rats of Groups 6 and 8 were exposed to chronic stressing. After this time all rats had an arterial catheter implanted. One day after implantation of the arterial catheter, the rats were connected to the BIOPAC system for recording MABP and HR at rest and during exposure to acute stress as in Groups 1–4. Subsequently, the rats of both groups were disconnected from the recording system and returned to their home cages where they had free access to food and water without captopril. After 24 h, measurements of MABP and HR at rest and during application of acute stress were repeated.

### 2.4. *Postmortem* Examination

After the experiments, the rats were killed by an overdose of pentobarbital (pentobarbital 10 mg/100 g of body wt, i.p.) and the heart was excised from the thorax. The wall of the left ventricle (including septum) was separated from the right ventricle and both atria. The infarct surface on the external and internal wall of the ventricle was measured and the measurements were averaged. The size of the infarct was determined planimetrically [31] with some modifications described previously [25] and expressed as a percentage of the total left ventricle surface. Fragments of the infarcted and noninfarcted left ventricle wall were harvested for histological verification of the presence of postinfarct fibrosis. The fragments were placed in a 4% formaldehyde solution and embedded in paraffin.

Tissue blocks were sectioned at 4 *μ*m and stained with hematoxilin and eosin for routine morphological examination [28, 30]. The abdomen was opened to check whether the end of the arterial catheter was located below the renal arteries and did not obstruct the lumen of the aorta or the renal artery.

### 2.5. Statistical Analysis

Statistical software (release 10) was used for statistical analysis of the results using recommendations of Curran-Everett and Benos [32] and Ludbrook [33] for independent and repeated measurements and the post hoc analysis of data. The Shapiro-Wilk test was used to check whether the data follow a normal distribution. Multiple-way ANOVA (two levels of rats: infarcted versus sham-operated; 2 levels of repeated measurements: before and after captopril intake; and 4 levels of experimental design: sham-operated nonchronically stressed, sham-operated chronically stressed, infarcted nonchronically stressed, and infarcted chronically stressed) was used to determine the significance of differences between the resting cardiovascular parameters and between the maximum increases, latencies, and durations of the cardiovascular parameters produced by the air jet stressor. The horizontal and vertical multiple pairwise comparisons were made using the post hoc Tukey test; *t*-test (paired or unpaired) was used if two groups of measurements were compared. The differences were considered significant if *P* was <0.05. Values presented in the text and figures correspond to means and standard errors.

## 3. Results

### 3.1. Effect of Captopril Intake on Resting MABP and HR

**Figure 2:**
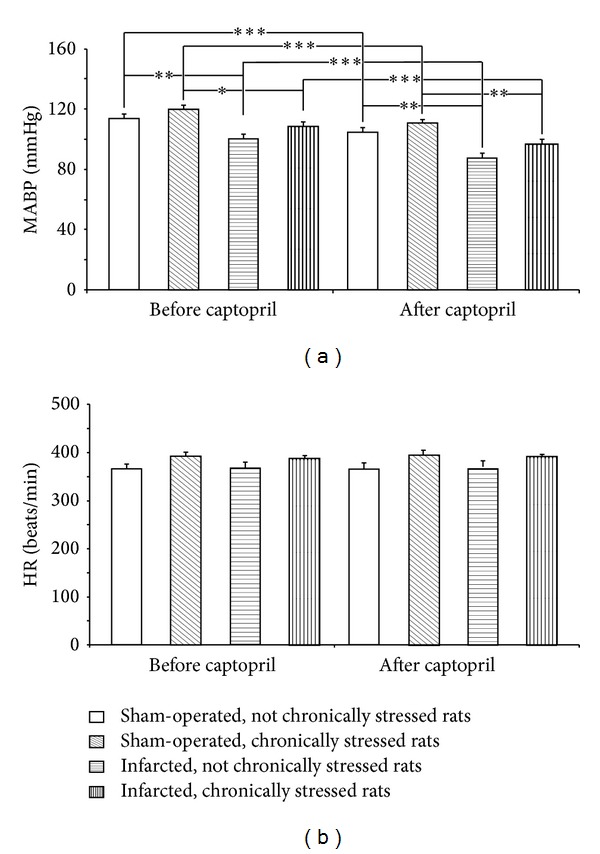
Changes in resting mean arterial blood pressure (MABP) and heart rate (HR) before and after captopril intake in the infarcted or sham-operated rats, exposed or not exposed to mild chronic stressing. Means ± SE are shown; *P < 0.05; **P < 0.01; ***P < 0.001.

Significant differences were found between the individual experimental groups both before [*F*(3.25) = 10.32; *P* < 0.001] and after captopril ingestion [*F*(3.25) = 13.47; *P* < 0.001]. The resting MABP was significantly lower in the rats drinking water containing captopril than in those drinking pure water. A comparison between the individual groups showed that before the administration of captopril the resting MABP was significantly lower in the infarcted nonchronically stressed rats than in the sham-operated nonchronically stressed rats (Figure 2). MABP was also lower in the infarcted chronically stressed rats than in the sham-operated chronically stressed rats (Figure 2).

There were no significant differences in the resting heart rate, which did not differ either before or after administration of captopril (Figure 2).

### 3.2. Effect of Captopril on Air Jet Stress-Induced Maximum Changes in MABP and HR

**Figure 3:**
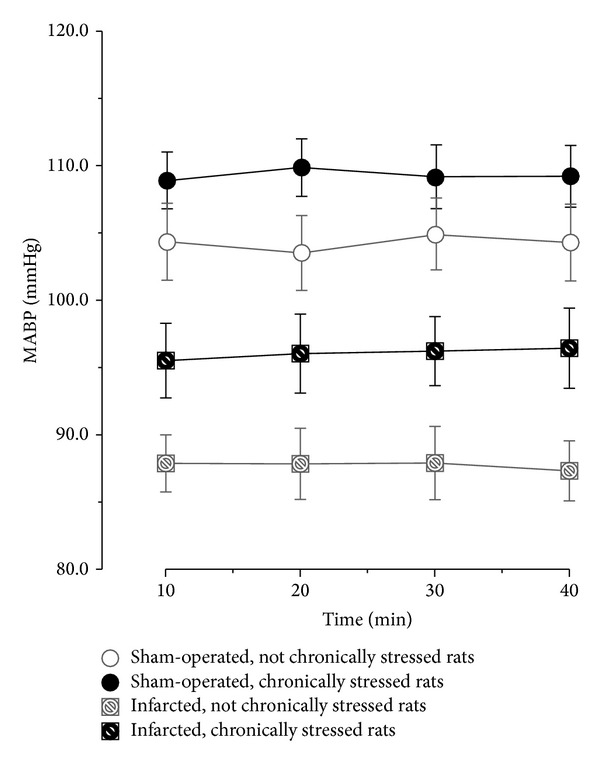
Fluctuations of baseline mean arterial blood pressure (MABP) during 40 min preceding application of the acute stress in the infarcted or sham-operated rats, exposed or not exposed to mild chronic stressing.

**Figure 4:**
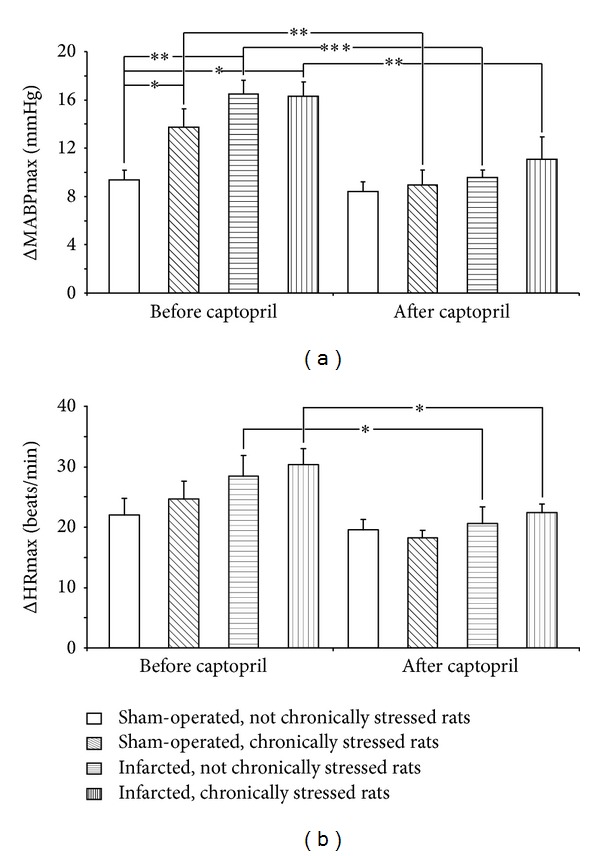
Maximum increases in mean arterial blood pressure (ΔMABPmax) and heart rate (ΔHRmax) after application of air jet stressor before and after captopril intake in the infarcted or sham-operated rats, exposed or not exposed to mild chronic stressing. Means ± SE are shown; *P < 0.05; **P < 0.01; ***P < 0.001.

As shown in Figure 3 the baseline MABP were stable during 40 min preceding application of the acute stress. The corresponding changes of HR were also not significant (data not shown). The acute stress elicited significant increases of MABP and HR (Figure 4). One-way ANOVA revealed that before ingestion of captopril significant differences in the magnitude of cardiovascular responses to stress were present between the individual experimental groups [*F*(3.25) = 6.61; *P* < 0.001]. Individual comparisons showed that ΔMABPmax values were significantly higher in the sham-operated chronically stressed, infarcted nonchronically stressed, and the infarcted chronically stressed rats than in the sham-operated nonchronically stressed rats (Figure 4).

Captopril decreased the air jet stress-induced ΔMABPmax in each experimental group except for the sham-operated Group 1 (Figure 4). Before captopril administration, the intergroup differences in ΔHRmax were not significant. Ingestion of captopril resulted in significant decrease of ΔHRmax in the infarcted nonchronically stressed rats and in the infarcted chronically stressed rats (*P* < 0.05) (Figure 4).

### 3.3. Duration and Latency of the Pressor and Tachycardic Responses to Air Jet Stressor

**Figure 5:**
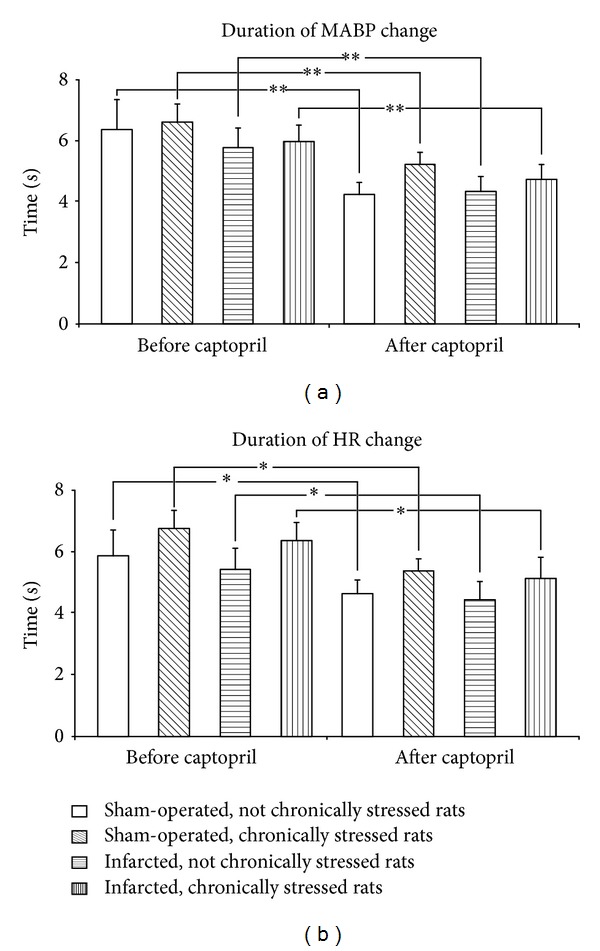
Duration of air jet stress-induced increases in mean arterial blood pressure (ΔMABP) and heart rate (ΔHR) before and after captopril intake in the infarcted or sham-operated rats, exposed or not exposed to mild chronic stressing. Means ± SE are shown; *P < 0.05; **P < 0.01.

A comparison of ΔMABP and ΔHR durations before and after administration of captopril by multiple-way repeated measures ANOVA revealed the presence of significant differences {ΔMABP: [*F*(1.25) = 6.69; *P* < 0.05]; ΔHR: [*F*(1.25) = 8.51; *P* < 0.05]} (Figure 5). Individual comparisons demonstrated that captopril reduced the duration of ΔMABP and ΔHR in each group (Figure 5).

**Figure 6:**
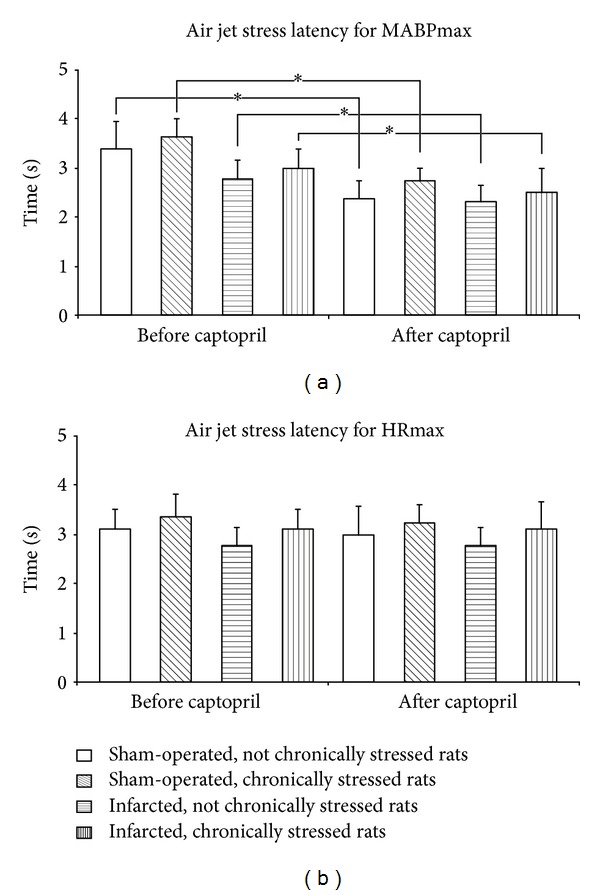
Latency of air jet stress-induced maximum increases in mean arterial blood pressure (ΔMABPmax) and heart rate (ΔHRmax) before and after captopril intake in the infarcted or sham-operated rats, exposed or not exposed to mild chronic stressing. Means ± SE are shown; *P < 0.05.

A comparison of the latencies to ΔMABPmax before and after administration of captopril by multiple-way repeated measures showed significant differences [*F*(1.25) = 13.92; *P* < 0.01]. As shown in Figure 6, the latencies to ΔMABPmax after administration of captopril were significantly lower than before administration of this compound. Administration of captopril did not affect latencies of ΔHRmax in any of the experimental groups (Figure 6).

#### 3.3.1. Supplementary Experiments (Groups 5–8)

**Figure 7:**
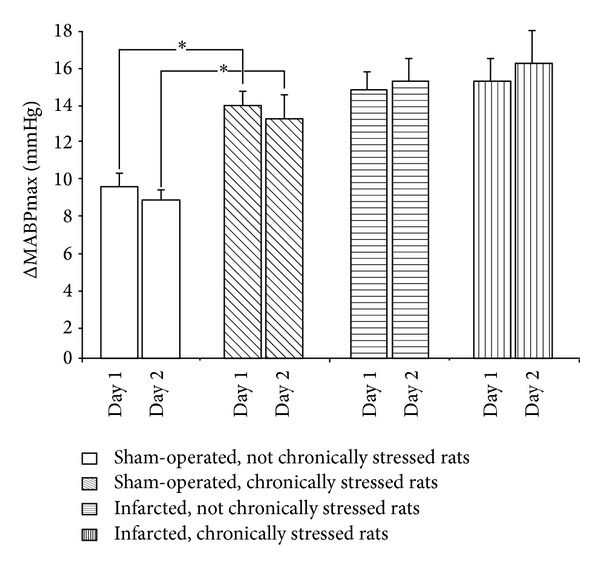
Maximum increases of blood pressure during application of air jet stress in the infarcted or sham-operated rats, exposed or not exposed to mild chronic stressing. Air jet stressor was applied twice with 24 h interval (day 1 and day 2). Means ± SE are shown; *P < 0.05.

As shown in Figure 7, acute stress produced the same maximum increases of MABP in the rats drinking water without captopril when it was repeated with 24 h intervals. Repeatability of responses was present both in the sham-operated and in the infarcted rats.

#### 3.3.2. Other Measurements

Left ventricle end-diastolic pressure in the infarcted, nonchronically stressed rats (24.88 ± 1.93) was significantly higher than in the sham-operated, nonstressed rats (3.88 ± 0.61; *P* < 0.001). Similarly, LVEDP in the infarcted, chronically stressed rats (22.56 ± 1.56) was significantly higher than in the sham-operated, chronically stressed rats (3.38 ± 0.63; *P* < 0.001). Left ventricle end-diastolic pressure in the infarcted, nonchronically stressed rats, receiving captopril (18.33 ± 0.95), was higher than in the sham-operated, nonstressed rats, receiving captopril (2.88 ± 0.35; *P* < 0.001). Similarly, LVEDP in the infarcted, chronically stressed rats, receiving captopril (16.38 ± 0.84), was significantly higher than in the sham-operated, chronically stressed rats, receiving captopril (2.75 ± 0.37; *P* < 0.001). Captopril significantly reduced LVED in all infarcted rats (*P* < 0.01) but did not exert significant effect on LVEDP in the sham-operated chronically stressed rats.

The* postmortem* measurements showed that the infarct surfaces were similar in the all-infarcted, nonchronically stressed rats (range 25–45%) and in the all-infarcted, chronically stressed rats (range 26–47.5%).

## 4. Discussion

A novel finding in the present study is that the oral administration of ACE inhibitors normalizes cardiovascular responses to acute stress in infracted and chronically stressed rats.

### 4.1. Hypotensive Effect of Short-Term Administration of Captopril on Resting Blood Pressure

In the present study, we decided to evaluate the effect of oral administration of captopril as it is the routine way for the application of ACE inhibitors during the treatment of cardiovascular diseases. In our study, the dose of captopril ingested in 24 h water supply was sufficient to decrease the resting blood pressure in all experimental groups, including the sham-operated nonstressed rats. The finding that captopril decreases the resting blood pressure in the infarcted and chronically stressed rats fits with the activation of the systemic and local renin-angiotensin systems during postinfarct heart failure [17, 34, 35] and during chronic stress [29]. For instance, Ahmad et al. [35] have found that systematically applied lisinopril—one of the hydrophilic ACE inhibitors—reduces ACE activity, both in the brain and in the kidney of the infarcted rats. A decrease of the resting blood pressure after captopril indicates that the dose of captopril, which was applied, was sufficient to exert the hypotensive effect in the control nondisturbed animals. Previously, a significant hypotensive effect was also observed after chronic application of captopril in conscious Sprague Dawley control rats [23] and after administration of 25 mg of this compound into healthy human subjects in an upright position [36].

### 4.2. Suppression of Exaggerated Cardiovascular Responses to Acute Stress by Captopril in Infarcted and Chronically Stressed Rats

Our results confirm previous findings showing enhanced cardiovascular responses to acute stress in myocardially infarcted rats [17, 18, 25, 30] and in chronically stressed rats [30]. In previous studies, it was demonstrated that this effect significantly depends on the activation of the brain renin-angiotensin system as it could be abolished by ICV administration of AT1 receptor blockers and potentiated by central administration of Ang II [17–19]. The present study shows that significant attenuation of exaggerated cardiovascular responses to acute stress may be achieved both in infarcted and in chronically stressed rats by oral administration of captopril.

It cannot be excluded that the suppression of cardiovascular responses to stress by captopril could result from the inhibition of the brain ACE. Indirect evidence indicates that peripheral administration of ACE inhibitors, including captopril, may inhibit the brain renin-angiotensin system [35, 37, 38]. Moreover, autoradiographic studies have shown that chronic oral administration of ACE inhibitor quinapril lowers ACE density in several brain structures by 35–38% [39]. On the other hand, there are also studies showing that Ang II modulates sympathoadrenal activity through peripheral action [1, 40]. Thus, the stress-suppressing inhibitory effect of captopril might be partly related to the suppression of the stimulation of the sympathoadrenal system, which is significantly stimulated both during postinfarct heart failure [17] and during chronic stressing [22, 29, 41, 42].

The present study shows that oral administration of captopril significantly reduces exaggerated cardiovascular responses to acute stress during chronic stressing. Previous studies provided evidence for pronounced activation of the renin-angiotensin system during stress. Thus, significant increases in Ang II content in the hypothalamus, medulla oblongata, heart, and adrenal medulla were found in the rats subjected to acute and prolonged stress by Yang et al. [9]. Moreover, it has been shown that a restraint stress enhances expression of AT1 receptors mRNA in the paraventricular nucleus (PVN) [43]. It was also demonstrated that AT1 receptors play a significant role in the activation of the hypothalamo-pituitary-adrenal axis during stress [10, 11, 13]. Previous studies showed that the blockade of central AT1 receptors in the rostral, ventrolateral medulla (RVLM) significantly attenuates pressor responses to short-lasting emotional stress in rabbits [15, 16]. In addition, Saiki et al. [14] reported that the blockade of central AT1 receptors suppresses stimulation of the sympathetic nervous system and reduces pressor and tachycardic responses elicited by immobilization stress. The present study implies the involvement of Ang II in the regulation of blood pressure during chronic stressing; however its action may be mediated by other compounds.

Our previous studies provided evidence for interaction of vasopressin and angiotensin II in the regulation of cardiovascular responses to acute stress. We have also found that vasopressin and stimulation of V1 receptors participate in Ang II-induced potentiation of the cardiovascular responses to acute stress in infarcted rats [18, 19]. Thus, reduction of the cardiovascular responses to acute stress by captopril could result from its inhibitory effect on generation of Ang II in vasopressin secreting neurons. In this line, it has been shown that inhibition of ACE by oral application of quinapril or ramipril reduces the content of vasopressin in the supraoptic and paraventricular (PVN) nuclei as well as in the brain regions innervated by vasopressin secreting neurons [39, 44].

Apart from inhibition of Ang II formation, ACE inhibitors may also act by activation of the kinins system and release of bradykinin [21, 45] and through the stimulation of nitric oxide synthase and 20-HETE pathway [46, 47].

Interestingly, captopril significantly shortened the duration of the pressor and tachycardic responses to the air jet stressor in all experimental groups and reduced the latency to the maximum increase in arterial blood pressure (Figures 5 and 6). Reduced duration of the pressor and tachycardic responses to acute stress may be considered as a positive result of ACE treatment because most likely it was associated with reduced cardiac workload. The shortening of the latency to the air jet stress-induced maximum elevation in MABP by captopril may suggest that the blockade of ACE enhances alertness to the alarming stressor.

In conclusion, the present study shows that orally administered captopril significantly decreases resting arterial blood pressure and significantly reduces the pressor and tachycardic responses to the acute stressor in chronically stressed rats and in infarcted rats exposed and not exposed to chronic stressing. Thus, the study discloses a new aspect of beneficial action of orally administered ACE inhibitors in the postinfarct cardiac failure and during chronic exposure to stress.
